# Effects of renal impairment on cardiac remodeling and clinical outcomes after myocardial infarction

**DOI:** 10.7150/ijms.61891

**Published:** 2021-06-01

**Authors:** Chun-Yen Chiang, Sheng-Chung Huang, Michael Chen, Jhih-Yuan Shih, Chon-Seng Hong, Nan-Chun Wu, Chung-Han Ho, Chia Chun Wu, Zhih-Cherng Chen, Wei-Ting Chang

**Affiliations:** 1 Division of Cardiology, Department of Internal Medicine, Chi Mei Medical Center, Tainan.; 2 Department of Optometry, Chung Hwa University of Medical Technology, Rende District, Tainan.; 3 Department of Health and Nutrition, Chia Nan University of Pharmacy and Science, Tainan.; 4 Division of Cardiovascular Surgery, Department of Surgery, Chi Mei Medical Center, Tainan.; 5 Department of Pharmacy, Chia Nan University of Pharmacy and Science, Tainan.; 6 Department of Hospital and Health Care Administration, Chi-Mei Medical Center, Tainan.; 7 Division of Nephrology, Department of Internal Medicine, Chi Mei Medical Center, Tainan.; 8 Department of Biotechnology, Southern Taiwan University of Science and Technology, Tainan.; 9 Institute of Clinical Medicine, College of Medicine, National Cheng Kung University, Tainan.

**Keywords:** post myocardial infarction, cardiac remodeling, renal function, mortality, heart failure

## Abstract

How renal function influences post-acute myocardial infarction (AMI) cardiac remodeling and outcomes remains unclear. This study evaluated the impact of levels of renal impairment on drug therapy, echocardiographic parameters, and outcomes in patients with AMI undergoing percutaneous coronary intervention (PCI). A total of 611 patients diagnosed with AMI underwent successful PCI, and two echocardiographic examinations were performed within 1 year after AMI. Patients were categorized according to Group 1: severely impaired estimated glomerular filtration rate (eGFR)<30, Group 2: mildly impaired 30≤eGFR<60, Group 3: potentially at risk 60≤eGFR<90 and normal eGFR≥90 ml/min/1.73 m2. During the 5-year follow-up period, the primary endpoints were cardiovascular mortality and outcomes. Patients with worse renal function (eGFR<30) were older and had a higher prevalence of hypertension and diabetes, but relatively few were smokers or had hyperlipidemia. Despite more patients with lesions of the left anterior descending artery, those with worse renal function received suboptimal guideline-directed medical therapy (GDMT). Notably, patients with worse renal function presented with worse left ventricular function at baseline and subsequent follow-up. Kaplan-Meier analysis revealed increased cardiovascular death, development of heart failure, recurrent AMI and revascularization in patients with worse renal function. Notably, as focusing on patients with ST elevation MI, the similar findings were observed. In multivariable Cox regression, impaired renal function showed the most significant hazard ratio in cardiovascular death. Collectively, in AMI patients receiving PCI, outcome differences are renal function dependent. We found that patients with worse renal function received less GDMT and presented with worse cardiovascular outcomes. These patients require more attention.

## Introduction

The incidence of renal impairment is growing, and the condition is not only a direct threat to health but also an important risk factor for cardiovascular disease 1-3. Among patients hospitalized for acute myocardial infarction (AMI), renal impairment is also an important predictor of cardiovascular complications, including heart failure, arrhythmia and coronary arterial restenosis 1, 4. Previous studies have mainly focused on patients with preserved renal function, while patients with renal impairment have either been undervalued or excluded from clinical trials 3, 4. Additionally, in consideration of comorbidities and contrast-induced nephropathy, patients with renal impairment may be delayed to revasculization or receive less optimal therapies for AMI than those with preserved renal function 3-6. Given advances in treatment, including prompt coronary reperfusion and guideline-directed medical therapy (GDMT), including antiplatelet agents, beta-blockers, statins and inhibitors of the renin-angiotensin-aldosterone system, the short-term morbidity and mortality for patients with AMI has improved, but the long-term effect on post-AMI cardiac remodeling and survival remains largely unknown 6. In this five-year longitudinal study, we comprehensively investigated the influence of renal function on drug therapy, post-AMI short- and long-term cardiac structure, cardiac function, and outcomes among AMI patients receiving percutaneous coronary intervention (PCI).

## Methods

### Study design

From January 2014 to December 2017, we retrospectively collected information on 643 hospitalized patients with AMI. The diagnosis was validated by two of the following three criteria: (1) history of prolonged ischemic chest pain, (2) evolutionary changes in the ST segment and/or T wave, and (3) dynamic elevation of serum cardiac enzymes such as cardiac troponin and/or creatine kinase-muscle/brain (MB). All studied patients received PCI as reperfusion therapy. Clinical information regarding comorbidities and prescriptions for cardiovascular medications were also obtained. Smoking was defined as current or former and hyperlipidemia as receiving lipid-lowering therapy or either total cholesterol >200 mg/dl or serum triglycerides >150 mg/dl 7. Serum creatinine and estimated glomerular filtration rate (eGFR) levels were obtained at the time of admission for AMI. Patients were categorized into three groups in accordance with their renal function as eGFR by the Cockcroft-Gault equation (Group 1: severely impaired eGFR<30, Group 2: mildly impaired 30≤eGFR<60, Group 3: potentially at risk 60≤eGFR<90 and Group 4: normal eGFR ≥ 90 ml/min/1.73m^2^). Patients, who were lost to follow-up, had a poor imaging window, died during the same hospitalization, received coronary bypass surgery or were under dialysis were excluded. Angiographic stenosis was defined as >50% stenosis, and indication for PCI was >70% narrowing of the coronary artery luminal diameter. The median follow-up duration was 41 (interquartile range: 22-60) months. The study was conducted in accordance with the tenets of the Declaration of Helsinki and approved by the Review Board of Chi-Mei Hospital (CV code: 10410-002).

### Echocardiographic parameters

In accordance with the recommendations of the American Society of Echocardiography 8, echocardiography was performed with a 3.5-MHz multiphase-array probe and GE Vivid E9 system (Vingmed Ultrasound AS, Horten, Norway) by the cardiologists in charge. The chamber dimensions (LVEDVi = left ventricular end diastolic volume index; LVESVi = left ventricular end systolic volume index) and left ventricular mass index (LVMi) were measured using the two-dimensionally guided M-mode method, and LV ejection fraction (LVEF) was measured using the biplane Simpson's method. In addition, LV diastolic function-associated parameters, including the transmitral early filling velocity (E) to atrial velocity (A) ratio, were also measured. Echocardiography was performed during hospitalization and within one year post-AMI to evaluate the subsequent changes in cardiac geometry and function.

### Outcomes

According to a medical review, the following clinical outcomes were analyzed: cardiovascular mortality, heart failure (HF) requiring hospitalization, recurrent AMI, ventricular arrhythmia, and coronary revascularization owing to stent thrombosis or restenosis. The diagnosis was validated by at least three cardiologists in a blinded manner.

### Statistical analysis

Continuous data are presented as the mean ± standard deviation or median (interquartile range), depending on the distribution. Dichotomous data are presented as numbers and percentages. The chi-squared test or Fisher's exact test was used to compare categorical variables as appropriate. Group differences were analyzed using analysis of variance, including post hoc analysis and multiple testing adjustment. The Kaplan-Meier method with log-rank test was used to compare event-free rates between groups. A univariate Cox regression analysis was performed to evaluate factors associated with mortality. Factors with *p* <0.1 based on univariate analysis were included in multivariable Cox regression analysis to identify independent risk factors for endpoints. Given that AMI includes ST elevation MI and non- ST elevation MI, as a sensitivity test, we also focused on the analyses of patients diagnosed of ST elevation MI to evaluate whether our findings are consistent in different populations. All analyses were performed using SPSS, version 18 for Windows (SPSS Inc., Chicago, IL, USA).

## Results

### Baseline characteristics of AMI patients receiving PCI

The final sample consisted of 611 patients. The average age of the patients was 71 years-old. Among them, 56% were men, and the majority (93%) had one or more cardiovascular risk factors, including hypertension, diabetes, hyperlipidemia and smoking. Notably, 150 of them had renal at potential risk of impairment (eGFR 60-90 mL/min/1.73 m), 216 of them had mild renal impairment (eGFR 30-60 mL/min/1.73 m), while 151 of them had severe renal impairment (eGFR<30 mL/min/1.73 m) at the time AMI was diagnosed (Table 1). Among patients with severe renal impairment, interestingly, we found more older and female patients with a higher prevalence of hypertension and diabetes but a relatively lower body mass index and less hyperlipidemia and smoking than the others. Regarding the coronary intervention, the complexity of CAD was similar among groups, but there were more interventions for LAD in patients with severe renal impairment.

### Post-AMI prescriptions among patients with different renal functions

Despite more significant lesions at LAD, it is noteworthy that before discharge, patients with renal impairment received less GDMT. Among patients with severe renal impairment, only 90%, 54.3%, 56.3% and 45.6% were prescribed antiplatelet agents, statins, angiotensin-converting enzyme inhibitors/angiotensin receptor blockers (ACEis/ARBs) and β-blockers, respectively. Conversely, in patients with preserved renal function, the prescription rates were much higher at 100%, 81.9%, 79.8% and 76%, respectively.

### Post-AMI cardiac structural and functional changes among patients with different renal functions

In terms of echocardiography-derived cardiac function during hospitalization, compared with patients with preserved renal function, there were significantly higher LV volumes (LVEDVi 62.1±19.2 ml/M2 vs. 71.9±27.9 ml/M2 vs. 83.4±37.5 ml/M2 vs. 91.9±44.1 ml/M2 of preserved renal function, potentially-at-risk, mild and severe renal impairments, respectively, p=0.001), mass index (LVMi 105.7±43.7 g/M2 vs. 127.8±54.8 g/M2 vs. 138.1±71.9 g/M2 vs. 164.5±85.2 g/M2 of preserved renal function, potentially-at-risk, mild and severe renal impairments, respectively*,* p=0.001) and lower LV systolic function (LVEF 57.2±20% vs. 56.1±24.5% vs. 57.4±21.4% vs. 52.5±23.1 of preserved renal function, potentially-at-risk, mild and severe renal impairment, respectively, p=0.01) among patients with renal impairment, especially those with severe renal impairment. In contrast, diastolic function did not show significant differences among groups. In the longitudinal follow-up, despite slight improvements in LV systolic function one year post AMI (changes of LVEF 3.7±17.3% vs. 2.9±44.4% vs. 3.8±20.1% vs. 3.8±23.6% of preserved renal function, potentially-at-risk, mild and severe renal impairment, respectively, p=0.01), the changes were not significantly different among groups. Notably, one year post AMI, the myocardial function in patients with impaired renal function remained lower than that in those with preserved renal function (LVEF 60.9±12.5% vs. 62.1±13.2% vs. 61.1±15% vs. 56.6±15.1 of preserved renal function, potentially-at-risk, mild and severe renal impairment, respectively, p=0.01).

### Survival and cardiovascular outcomes among patients with different levels of renal function

**Figure 1:**
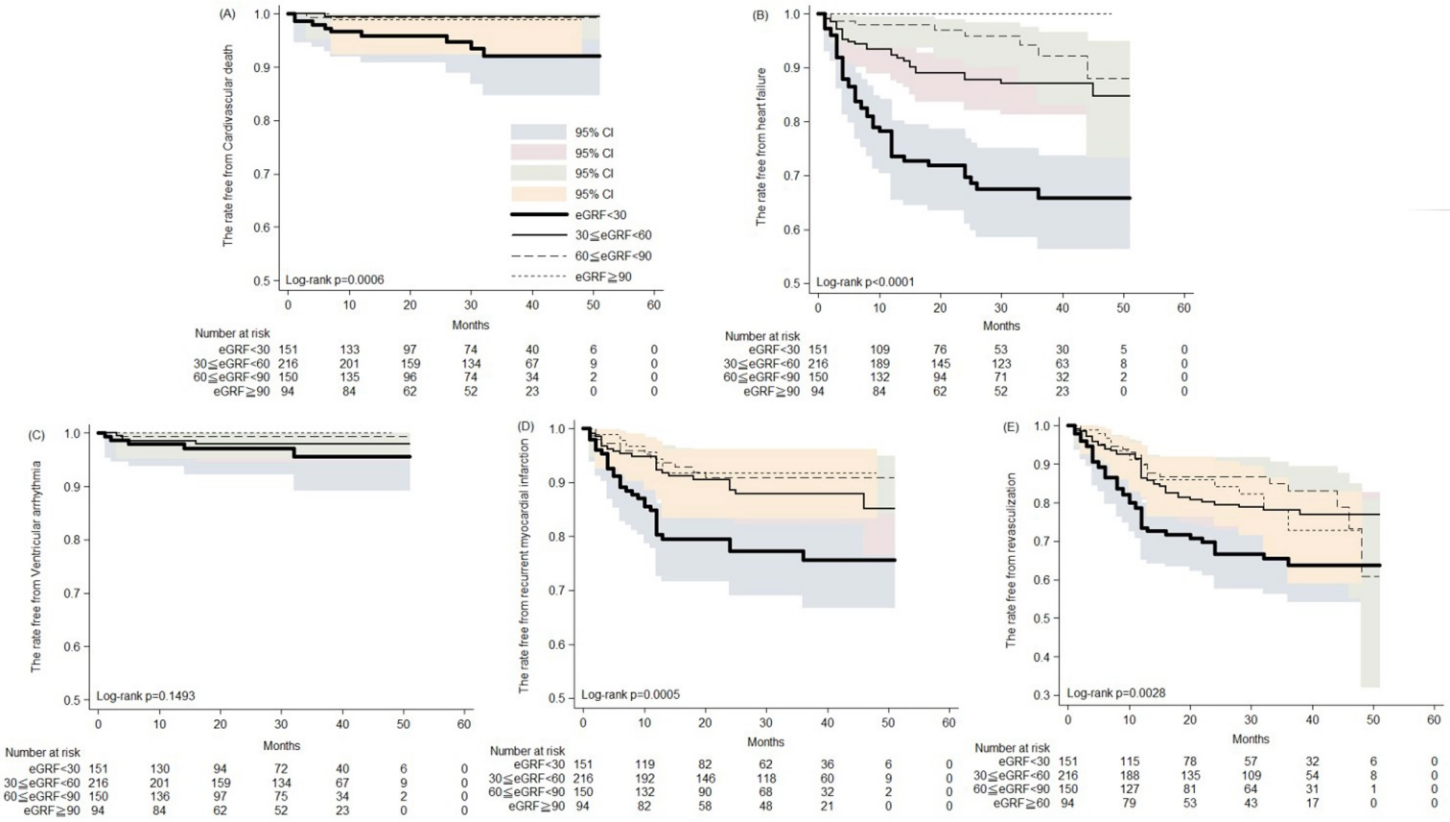
The Kaplan-Meier analysis of the rates free from (A) cardiovascular death; (B) heart failure hospitalization; (C) ventricular arrhythmia; (D) recurrent myocardial infarction; (E) revasculization in patients of acute myocardial infarction receiving percutaneous coronary intervention.

During the five years follow-up period, the Kaplan-Meier analysis revealed increased cardiovascular death, development of heart failure, recurrent MI and revascularization in patients with severely impaired renal function (Figure 1). Thirty to 50 months post AMI, the rates free from CV death were 93.3% and 91.9%, respectively, in patients with severe renal impairment, compared with 98.9% and 98.9% in those with preserved renal function (Figure 1A). Likewise, 30 and 50 months post AMI, the rates free from recurrent AMI were 77.1% and 75.3%, respectively, in patients with severe renal impairment, compared with 91.7% in those with preserved renal function (Figure 1D). Post AMI at 30 and 50 months, rates free from coronary revasculization were 66.4% and 63.4%, respectively, in patients with severe renal impairment, compared with 82.2% and 72.5% in those with preserved renal function (Figure 1E). Notably, among patients with preserved renal function, none developed HF that required hospitalization. In contrast, among patients with severe renal impairment the rates free from HF hospitalization were only 67.3% and 65.5% at the 30th and 50th months post AMI, respectively (Figure 1B). Rates of post AMI fatal arrhythmia, such as VT and VF, were not significantly different among patients with various levels of renal function.

### Clinical risk factors contributing to cardiovascular mortality

To further compare the impact of renal impairment with other clinical risk factors for cardiovascular adverse events, using Cox regression we found that BMI, DM, HTN, severe renal impairment (Group 1) LAD lesions, LVEF and LVESVi one year post AMI were significantly associated with CV death (Table 2). Interestingly, in the multivariable analysis only severe renal impairment significantly correlated to CV death (HR 4.87, CI: 1.24-19.02, p=0.02). Collectively, our findings highlighted the importance of impaired renal function at the diagnosis of AMI. Patients with impaired renal function not only received less GDMT but had worse post-MI cardiac remodeling and long-term cardiovascular outcomes.

### Analyses focusing on ST elevation MI

To evaluate whether our findings are consistent in different populations, we focused on the analyses of 126 patients diagnosed of ST elevation MI (Supplement Table 1). Interestingly, we also found that patients with worse renal function (eGFR<30) were older and had a higher prevalence of hypertension and diabetes, but relatively few were smokers or had hyperlipidemia. Likewise, despite more multi-vessel coronary arterial diseases, especially lesions of the left anterior descending artery, in patients with worse renal function, they received less optimal GDMT. In term of myocardial function, patients with worse renal function presented with worse left ventricular function at baseline and subsequent follow-up. Although not statistically significant, there were relatively more cardiovascular death, development of heart failure in patients with severe renal impairment compared with others.

## Discussion

In comparison to patients with preserved renal function, those with renal impairment were more likely to be older and have a greater prevalence of comorbidities 9, 10. Additionally, in consideration of drug-related adverse effects such as hyperkalemia, patients with impaired renal function were less likely to be prescribed ACEIs and ARBs during hospitalization 11, 12. However, surprisingly, not only renin-angiotensin-aldosterone inhibitors but also other GDMTs, including antiplatelet agents, statins and beta-blockers, were less prescribed. Despite a marked reduction of in-hospital death in patients with renal impairment who received coronary interventional procedures 13, Sattar et al reported that among patients with severe renal impairment, PCI was performed with less complex anatomy on account of the risks of contrast-induced nephropathy 14. In a multicenter PCI registry, patients with renal impairment were more likely to develop heart failure, atrial fibrillation, and cardiogenic shock during hospitalization than patients with preserved renal function 15. Additionally, the severity of renal dysfunction was associated with an increased risk of all‐cause mortality 9, 14, 16. Similarly, we observed that in AMI patients receiving PCI, those with renal impairment received less GDMT even if they had stenotic lesions on the left anterior descending artery. Through a longitudinal follow-up of echocardiography, we found that patients with worse renal function had worse left ventricular function during hospitalization and one year post AMI. Most importantly, cardiovascular outcome in AMI patients receiving PCI was significantly dependent on renal functions, while those with severe renal impairment showed the highest risks of cardiovascular death, HF hospitalization, coronary revasculization and recurrent AMI. Our findings highlighted the importance of aggressive management in patients with impaired renal function post AMI.

The complex pathophysiological interplay between heart and kidney diseases, also defined as cardiorenal syndrome, remains under investigation 2, 17. Previous studies suggested that renal disorder results in ventricular dysfunction and deteriorates remodeling after AMI through excessive renin-angiotensin activation 18. As kidney function declines, numerous metabolic pathways are disturbed, including altered volume and pressure status, accelerated atherosclerosis and arteriosclerosis, disturbed mineral metabolism, uremic toxins and oxidative stress, all of which stress cardiac cells 2, 18. Left ventricular hypertrophy, fibrosis and dysfunction trigger not only heart failure but also electric instability and sudden cardiac death 2, 19. From another perspective, renal impairment increases neointimal growth after percutaneous coronary intervention, resulting in higher rates of recurrent restenosis 20. Previous studies also indicated that renal impairment is significantly associated with an increased incidence of 1-year stent thrombosis in patients undergoing PCI 15, 21, 22.

It is noteworthy that the prevalence of coronary arterial disease in patients with renal impairment is nearly 70%, and vice versa, there is also a high ratio of AMI patients with renal dysfunction 3, 23. In our study, 84% of AMI patients had various degrees of renal impairment. Among them, a quarter had severe renal dysfunction, underscoring the importance of identifying renal impairment in patients diagnosed with AMI. Although patients with renal impairment were less likely to receive effective cardiac medications or undergo optimal coronary interventional procedures, prompt and effective strategies of coronary reperfusion and subsequent myocardial remodeling have been found to ameliorate the outcomes.

There are some limitations of this study. First, in a retrospective study design, the endpoints were not collected at the same time window after the initial admission while it may introduce a bias in the assessed outcomes. Second, given the limited number of patients diagnosed of ST elevation MI, the Cox regression analysis was not performed in this population. Despite abovementioned limitations, our research indicates an urgent need to improve the quality of care in patients with concomitant AMI and renal impairment 13.

## Conclusions

Among patients hospitalized for AMI, renal impairment not only interferes with the clinical decision of GDMT and coronary interventions but also plays a pivotal role in post-AMI cardiac remodeling and cardiovascular outcomes. Our findings highlight the need to pay more attention to AMI patients with renal impairment.

## Supplementary Material

Supplementary table.

## Figures and Tables

**Table 1:** The baseline clinical characteristics and sequential echocardiographic parameters in regard to renal function in patients with acute myocardial infarction (AMI) including both ST-elevation MI and non-ST elevation MI (N=611)

	Group 1 (<30 mL/min/1.73m) N=151	Group 2 (30-60 mL/min/1.73m) N=216	Group 3 (60-90 mL/min/1.73m) N=150	Group 4 (>90 mL/min/1.73m) N=94	P-value
Age	73.5±10.7^b,c^	70.3±9.9^ b,c^	59.2±10^a,d^	50.81±9.9^ a,d^	0.001
Gender (Male)	85 (56.3)^ b,c^	161 (74.5)^d^	129 (86)^d^	84 (89.3)^d^	<0.001
BMI	23±3.6^b^	24.1±3.8	25.4±3.3	32.6±35.7^a,b,d^	0.001
**Cardiovascular risk factors**					
Diabetes	85 (56.3)^ a,b,c^	76 (35.1)^ d^	50 (33.3)^ d^	34 (36.2)^ d^	0.001
Hypertension	91 (60.2)^ c^	129 (59.7)^ c^	84 (56)^ c^	11 (11.7)	<0.001
Hyperlipidemia	89 (58.9)^ c^	165 (76.3)^ d^	109 (72.6)^ d^	84 (89.4)^ d^	0.001
Smoking	55 (36.4)^ a,b,c^	100 (46.3)^ d^	77 (51.3)^ d^	46 (48.9)^ d^	0.005
**PCI for:**					
LAD	107 (70.8)^ a,b,c^	113 (52.3)^ d^	76 (50.6)^ d^	41 (43.6)^ d^	0.001
LCX	62 (41)	75 (34.7)	48 (32)	37 (39.4)	0.34
RCA	69 (45.6)^b,c^	116 (53.7)	92 (61.3)^ d^	65 (69.1)^ d^	0.002
One-vessel-disease	80 (52.9)	138 (63.8)	91 (60.6)	56 (59.5)	0.74
Two-vessel-disease	55 (36.4)	68 (31.5)	52 (34.6)	27 (28.7)	0.79
Three-vessel-disease	16 (10)	10 (4.6)	7 (4.6)	11 (11.7)	0.45
**Drugs before discharge**					
Anti-platelet agents	136 (90)^ a,b,c^	211 (97.7)^ d^	147 (98)^ d^	94 (100)^ d^	0.001
Statin	82 (54.3)^ a,b,c^	152 (70.4)^ d^	118 (78.6)^ d^	77 (81.9)^ d^	0.001
ACEIs/ARBs	85 (56.3)^ a,b,c^	152 (70.4)^ d^	129 (86)^ d^	75 (79.8)^ d^	0.001
β-blockers	69 (45.6)^ a,b,c^	130 (60.2)	87 (58)	63 (76)^ d^	0.005
***Echocardiographic parameters***					
**Post MI (during hospitalization)**					
LVEF (%)	52.5±23.1^a,b,c^	57.4±21.4^ d^	56.1±24.5^ d^	57.2±20^ d^	0.01
LVEDVi (ml/M2)	93.9±39.4^ a,b^	83.4±37.5^ c^	71.9±27.9^ c^	61.3±20.6^ d^	0.001
LVESVi (ml/M2)	44.5±25.5^ b,c^	35.7±25.4^ c,d^	28.2±17.9^ c,d^	26.2±13.6^ d^	0.001
LVMi (g/M2)	164.5±85.2^ a,b,c^	138.1±71.9 ^c,d^	127.75±54.8^ c,d^	105.7±43.7^ d^	0.001
E/A	1.1±0.5	1.04±0.5	1.06±0.7	1.1±0.6	0.96
E/e'	13.6±7.5	13.5±5.4	11.97±6	12.2±4.8	0.95
**Post MI (after one year)**					
LVEF (%)	56.6±15.1^ a^	61.1±15	62.1±13.2^ d^	60.9±12.5 ^d^	0.01
LVEDVi (ml/M2)	91.9±44.1^ a,b^	83.7±37.8	70.9±30.1^ d^	62.1±19.2^ d^	0.001
LVESVi (ml/M2)	43.7±29.9^ a,b^	35.5±25.5	29.5±20.7^ d^	25.7±14.9^ d^	0.001
LVMi (g/M2)	149.8±78.5^ a,b^	135.5±68.3	116.9±57.2^ d^	97.1±44.8^ d^	0.001
E/A	0.9±0.5	1.1±0.9	0.9±0.5	0.8±0.5	0.73
E/e'	14.2±6.9	12.5±5.2	12.3±6.4	11.7±5.3	0.12
Change of LVEF (%)	3.8±23.6	3.8±20.1	2.9±44.4	3.7±17.3	0.56
Change of LVMi (g/M2)	-14.5±98.8	-2.5±68.3	-10.8±57.1	-9.7±50.4	0.45
**Outcomes**					
Cardiovascular death	9	1	1	1	0.001
Time to death (months, median (IQR))	13 (8,25)			7	
Hear failure	45	26	8	0	0.001
Time to HF (months, median (IQR))	11 (7,25)				
Ventricular arrhythmia (VT/VF)	5	4	1	0	0.16
Time to VT/VF (months, median (IQR))	10 (1, 22)				
Recurrent myocardial infarction (MI)	32	24	12	7	0.001
Time to AMI (months, median (IQR))	8 (2, 20)			9 (4, 13)	
Revasculization	47	43	23	18	0.04
Time to revasculization (months, median (IQR))	12 (4, 26)			9 (1, 23)	

^a^*P <*0.05, compared with Group2; ^b^*P <*0.05, compared with Group3; ^c^*P <*0.05, compared with Group 4; ^d^*P <*0.05, compared with Group 1. BMI: body mass index; PCI: percutaneous coronary intervention; LAD: left anterior descending artery; LCX: left circumflex artery; RCA: right coronary artery; ACEIs/ARBs: angiotensin-converting enzyme inhibitors/angiotensin receptor blockers; LVEF: left ventricular ejection fraction; LVEDVi: left ventricular end diastolic volume index; LVESVi: left ventricular end systolic volume index; LVMi: left ventricular mass index; E/A: trans-mitral valve E to A velocity ratio; E/e′: mitral early filling velocity to early diastolic mitral annular velocity ratio; VT/VF: ventricular tachycardia/ventricular fibrillation.

**Table 2:** The univariate and multivariable hazard ratios of cardiovascular mortality in acute myocardial infarction (AMI) patients receiving percutaneous coronary intervention (PCI)

	Univariate HR (95% CI)	p	Multivariable HR (95% CI)	p
Age	1.01(0.96-1.04)	0.92		
Gender (Male)	2.6(0.87-7.74)	0.08		
BMI	0.91(0.83-1)	0.05	0.87(0.73-1.03)	0.1
DM	5.12(1.41-18.6)	0.01	1.04(0.19-5.44)	0.96
HTN	8.35(1.08-64.2)	0.04	6.01(0.7-50.98)	0.1
Hyperlipidemia	0.66(0.20-2.15)	0.49		
Severe renal impairment (Group 1)	9.59(2.59-35.43)	0.001	4.87(1.24-19.02)	0.02
LAD	9.95(1.29-76.53)	0.02	7.02(.61-80.81)	0.12
LCX	1.44(0.48-4.3)	0.5		
RCA	0.73(0.24-2.17)	0.57		
Anti-platelet agents	0.47(0.06-3.65)	0.47		
Statin	0.48(0.16-1.45)	0.19		
ACEi/ARB	0.47(0.15-1.4)	0.17		
Β-blockers	0.61(0.20-1.8)	0.37		
LVEF (%) (during hospitalization)	0.99(0.97-1.02)	0.87		
LVEDVi (ml/M2) (during hospitalization)	1.01(0.99-1.02)	0.33		
LVESVi (ml/M2) (during hospitalization)	1.01(0.98-1.02)	0.58		
LVMi (g/M2) (during hospitalization)	1(0.99-1)	0.98		
LVEF (%) (after one year)	0.96(0.92-0.99)	0.03	0.97(0.93-1.008)	0.12
LVEDVi (ml/M2) (after one year)	1.009 (0.99-1.02)	0.15	1.01 (0.99-1.06)	0.41
LVESVi (ml/M2) (after one year)	1.01(1-1.03)	0.03	0.99(0.98-1.001)	0.1
LVMi (g/M2) (after one year)	0.99(0.99-1.008)	0.81		

Abbreviation as listed in Table 1.
