# Supplementary material for: Effects of renal impairment on cardiac remodeling and clinical outcomes after myocardial infarction
Source: Int J Med Sci. 2021 Jun 1;18(13):2842–8. doi: 10.7150/ijms.61891 (PMC8241772; doi:10.7150/ijms.61891)
Supplement: Supplementary file 1 — Supplementary table. [file ijmsv18p2842s1.pdf]

**Supplement Table 1.** The baseline clinical characteristics and sequential echocardiographic parameters in regard to renal function in patients with ST elevation myocardial infarction (STEMI) (N=126)

| | Group 1 (<30 mL/min/1.73m) N=29 | Group 2 (30-60 mL/min/1.73m) N=59 | Group 3 (60-90 mL/min/1.73m) N=28 | Group 4 (>90 mL/min/1.73m) N=10 | P-value |
|---|---|---|---|---|---|
| Age | 76.5±11.4 [b,c] | 69±0.9 [b,c] | 57.8±11.1[d] | 47.1±9.82[d] | 0.001 |
| Gender (Male) | 15 (51.7) [a,b,c] | 51 (86.4) [d] | 26 (92.8) [d] | 8 (80) [d] | <0.001 |
| BMI | 23.1±3.67[c] | 23.5±3.5 [c] | 26.1±3.25 [c] | 34.8±3.5 [a,b,d] | 0.001 |
| Cardiovascular risk factors | | | | | |
| Diabetes | 18 (62.1)[a,b] | 14 (23.7)[d] | 7 (25) [d] | 4 (40) [d] | 0.003 |
| Hypertension | 22 (75.8) [b,c] | 35 (59.3) | 13 (46.4) | 3 (30) [d] | 0.03 |
| Hyperlipidemia | 19 (65.5) | 50 (84.7) | 25 (89.2) | 10 (100) | 0.12 |
| Smoking | 11 (37.9) | 27 (45.7) | 12 (42.8) | 4 (40) | 0.19 |
| PCI for | | | | | |
| LAD | 24 (82.7) [a,b,c] | 32 (54.2) [d] | 14 (50) [d] | 4 (40) [d] | 0.02 |
| LCX | 7 (24.1) | 23 (38.9) | 7 (25) | 1 (10) | 0.17 |
| RCA | 15 (51.7) [c] | 25 (42.4) [c] | 12 (42.8) [c] | 9 (90) [a,b,d] | 0.04 |
| One-vessel-disease | 16 (55.2) [b,c] | 39 (66.1) | 23 (82.1) [d] | 7 (70) [d] | 0.04 |
| Two-vessel-disease | 9 (31) | 19 (32.2) | 5 (17.8) | 2 (20) | 0.78 |
| Three-vessel-disease | 4 (13.7) | 1 (1.69) | 0 | 1 (10) | 0.87 |
| Drugs before discharge | | | | | |
| Anti-platelet agents | 27 (93.1) | 59 (100) | 28 (100) | 10 (100) | 0.07 |
| Statin | 14 (48.3) [a,b,c] | 46 (77.9) [d] | 24 (85.7) [d] | 10 (100) [d] | 0.001 |
| ACEIs/ARBs | 24 (82.7) | 51 (86.4) | 27 (96.4) | 9 (90) | 0.42 |
| β-blockers | 13 (44.8) | 39 (66.1) | 20 (71.4) | 6 (60) | 0.16 |
| **Echocardiographic parameters** | | | | | |
| Post MI (during hospitalization) | | | | | |
| LVEF (%) | 49.2±38.3 [a,b,c] | 62.5±14.3 [d] | 65.9±12.2 [d] | 64.3±7.7 [d] | 0.018 |
| LVEDVi (ml/M2) | 103±40.5 [b,c] | 95.1±37 | 78.7±27 [d] | 73.1±22 [d] | 0.001 |
| LVESVi (ml/M2) | 49.3±27.5 [b,c] | 38±26.56 [d] | 27.3±15.2 [d] | 26.3±10.7 3 [d] | 0.001 |
| LVMi (g/M2) | 161.3±84.4 [b,c] | 150.6±67 [d] | 130.8±42.2 [d] | 128.7±44.8 [d] | 0.001 |
| E/A | 1.1±0.6 | 1.1±0.5 | 1.1±0.6 | 1±0.4 | 0.99 |
| E/e’ | 13.7±7.9 | 13.5±5.4 | 12.2±5.1 | 13.4±5.1 | 0.76 |
| Post MI (after one year) | | | | | |
| LVEF (%) | 52.1±15.1 [a,b,c] | 64.7±13.8 [d] | 65.1±14 [d] | 68.4±11.7 [d] | 0.012 |
| LVEDVi (ml/M2) | 100.9±43.6 | 92.9±38.6 | 79.1±28.9 | 71±14.8 | 0.05 |
| LVESVi (ml/M2) | 40.7±27.1 | 34.9±25.8 | 28.4±18.5 | 22.5±13.4 | 0.18 |
| LVMi (g/M2) | 137.4±91.2 | 135.6±68.4 | 135.2±47.7 | 126.9±54 | 0.45 |
| E/A | 0.9±0.5 | 1.1±1.2 | 0.9±0.3 | 0.6±0.3 | 0.73 |
| E/e’ | 15.3±6.8 | 12.1±4.7 | 13.3±6.6 | 12.3±5 | 0.12 |

| | | | | | |
|---|---|---|---|---|---|
| Change of LVEF (%) | 5.9±29.4 | 2.1±13.8 | -0.7±10.9 | 4.1±10 | 0.29 |
| Change of LVMi (g/M2) | -23.8±83.1 | -14.9±59.5 | -4.3±52.6 | -1.7±75.8 | 0.14 |
| **Outcomes** | | | | | |
| Cardiovascular death | 2 | 0 | 0 | 0 | 0.07 |
| Hear failure | 8 | 6 | 3 | 0 | 0.05 |
| Ventricular arrhythmia (VT/VF) | 1 | 1 | 0 | 0 | 0.63 |
| Recurrent myocardial infarction (MI) | 5 | 6 | 2 | 2 | 0.5 |
| Revasculization | 8 | 13 | 7 | 3 | 0.92 |

[a]*P* <0.05, compared with Group2; [b]*P* <0.05, compared with Group3; [c]*P* <0.05, compared with Group 4; [d]*P* <0.05, compared with Group 1

Abbreviations as listed in Table 1
